# Study of effect of nimodipine and acetaminophen on postictal symptoms in depressed patients after electroconvulsive therapy (SYNAPSE)

**DOI:** 10.1186/s13063-022-06206-y

**Published:** 2022-04-18

**Authors:** Joey P. A. J. Verdijk, Julia C. M. Pottkämper, Esmée Verwijk, Guido A. van Wingen, Michel J. A. M. van Putten, Jeannette Hofmeijer, Jeroen A. van Waarde

**Affiliations:** 1 Department of Psychiatry, Rijnstate Hospital, Wagnerlaan 55, 6815 AD Arnhem, The Netherlands; 2 Clinical Neurophysiology, Institute for Technical Medicine, University of Twente, Technical Medical Centre, Hallenweg 15, 7522NB, Enschede, The Netherlands; 3 Department of Neurology, Rijnstate Hospital, Wagnerlaan 55, 6815 AD Arnhem, The Netherlands; 4 Department of Medical Psychology, Amsterdam University Medical Centres, location AMC, Meibergdreef 9, 1105 AZ Amsterdam, The Netherlands; 5 Department of Psychiatry, and Spinoza Centre for Neuroimaging, Amsterdam University Medical Centres, location AMC, Meibergdreef 9, 1105 AZ Amsterdam, The Netherlands

**Keywords:** Postictal, Acetaminophen, Nimodipine, Depression, Epilepsy, Electroconvulsive therapy, Electroencephalography, Arterial spin labelling (or perfusion weighted imaging), Cerebral perfusion, PROBE design

## Abstract

**Background:**

Postictal phenomena as delirium, headache, nausea, myalgia, and anterograde and retrograde amnesia are common manifestations after seizures induced by electroconvulsive therapy (ECT). Comparable postictal phenomena also contribute to the burden of patients with epilepsy. The pathophysiology of postictal phenomena is poorly understood and effective treatments are not available. Recently, seizure-induced cyclooxygenase (COX)-mediated postictal vasoconstriction, accompanied by cerebral hypoperfusion and hypoxia, has been identified as a candidate mechanism in experimentally induced seizures in rats. Vasodilatory treatment with acetaminophen or calcium antagonists reduced postictal hypoxia and postictal symptoms. The aim of this clinical trial is to study the effects of acetaminophen and nimodipine on postictal phenomena after ECT-induced seizures in patients suffering major depressive disorder. We hypothesize that (1) acetaminophen and nimodipine will reduce postictal electroencephalographic (EEG) phenomena, (2) acetaminophen and nimodipine will reduce magnetic resonance imaging (MRI) measures of postictal cerebral hypoperfusion, (3) acetaminophen and nimodipine will reduce clinical postictal phenomena, and (4) postictal phenomena will correlate with measures of postictal hypoperfusion.

**Methods:**

We propose a prospective, three-condition cross-over design trial with randomized condition allocation, open-label treatment, and blinded end-point evaluation (PROBE design). Thirty-three patients (age > 17 years) suffering from a depressive episode treated with ECT will be included. Randomly and alternately, single doses of nimodipine (60 mg), acetaminophen (1000 mg), or water will be given two hours prior to each ECT session with a maximum of twelve sessions per patient. The primary outcome measure is ‘postictal EEG recovery time’, expressed and quantified as an adapted version of the temporal brain symmetry index, yielding a time constant for the duration of the postictal state on EEG. Secondary outcome measures include postictal cerebral perfusion, measured by arterial spin labelling MRI, and the postictal clinical ‘time to orientation’.

**Discussion:**

With this clinical trial, we will systematically study postictal EEG, MRI and clinical phenomena after ECT-induced seizures and will test the effects of vasodilatory treatment intending to reduce postictal symptoms. If an effect is established, this will provide a novel treatment of postictal symptoms in ECT patients. Ultimately, these findings may be generalized to patients with epilepsy.

**Trial registration:**

Inclusion in SYNAPSE started in December 2019. Prospective trial registration number is NCT04028596 on the international clinical trial register on July 22, 2019.

## Administrative information

Note: the numbers in curly brackets in this protocol refer to SPIRIT checklist item numbers. The order of the items has been modified to group similar items (see http://www.equator-network.org/reporting-guidelines/spirit-2013-statement-defining-standard-protocol-items-for-clinical-trials/).

**Table:** 

Title {1}	StudY of effect of Nimodipine and Acetaminophen on Postictal Symptoms in depressed patients after Electroconvulsive therapy (SYNAPSE)
Trial registration {2a and 2b}.	Clinicaltrials.gov; identifier: NCT04028596 Date of registration: 19 July 2019
Protocol version {3}	07-01-2022, version 1.8
Funding {4}	Dutch Epilepsy Foundation
Author details {5a}	Rijnstate Hospital, Department of Psychiatry, Wagnerlaan 55, 6815 AD, Arnhem, The Netherlands; Rijnstate Hospital, Department of Neurology, Wagnerlaan 55, 6815 AD, Arnhem, The Netherlands; Amsterdam University Medical Centre, location AMC, Departments of Psychiatry, Medical Psychology and Spinoza Centre for Neuroimaging, Meibergdreef 9, 1105 AZ Amsterdam, The Netherlands; University of Twente, Technical Medical Centre, Department of Clinical Neurophysiology, Institute for Technical Medicine, Hallenweg 15, 7522NB, Enschede, The Netherlands.
Name and contact information for the trial sponsor {5b}	University of Twente, represented by C.H.G. Schoonheijt-Oude Veldhuis (Director of the Faculty Science and Technology).
Role of sponsor {5c}	The sponsor and funding party have no role in study management, analysis, interpretation of data, writing and submission. The research group is independent in the execution of the trial and has ultimate authority over these activities.

## Introduction

### Background and rationale {6a}

#### Postictal phenomena: burden in electroconvulsive therapy and epilepsy

In electroconvulsive therapy (ECT), seizures are induced to treat various psychiatric disorders, mostly pharmacotherapy-resistant mood disorders [1, 2]. Side effects of ECT include postictal phenomena such as confusion, delirium, headache, nausea, myalgia, and anterograde and retrograde amnesia [3]. These postictal phenomena cause a significant burden for patients receiving ECT and their relatives. Also, these symptoms may cause premature cessation or failure of this otherwise highly effective treatment. Moreover, postictal phenomena may impact the patient due to perceived stigma and rejection.

Patients with epilepsy suffer from reoccurring unpredictable seizures. The postictal state in patients suffering epilepsy is strikingly similar to those of ECT patients [4]. In patients with epilepsy, postictal phenomena may present as unresponsiveness, sensory, motor, or memory deficits, impaired cognition, headache, delirium, or psychosis, which may last several minutes to hours [5–8]. All postictal manifestations add to the morbidity of epilepsy and have been appointed as a neglected entity in the management of seizures [6]. For patients and their relatives, postictal phenomena enhance the burden of epilepsy.

Given the erratic nature of epileptic seizures, investigating the postictal state is not straightforward [8]. The predictability of ECT-induced seizures, on the other hand, offers a unique opportunity to examine postictal phenomena systematically. In humans, though, epileptologic studies in ECT patients are very limited.

#### ECT as a human seizure model

**Fig. 1:**
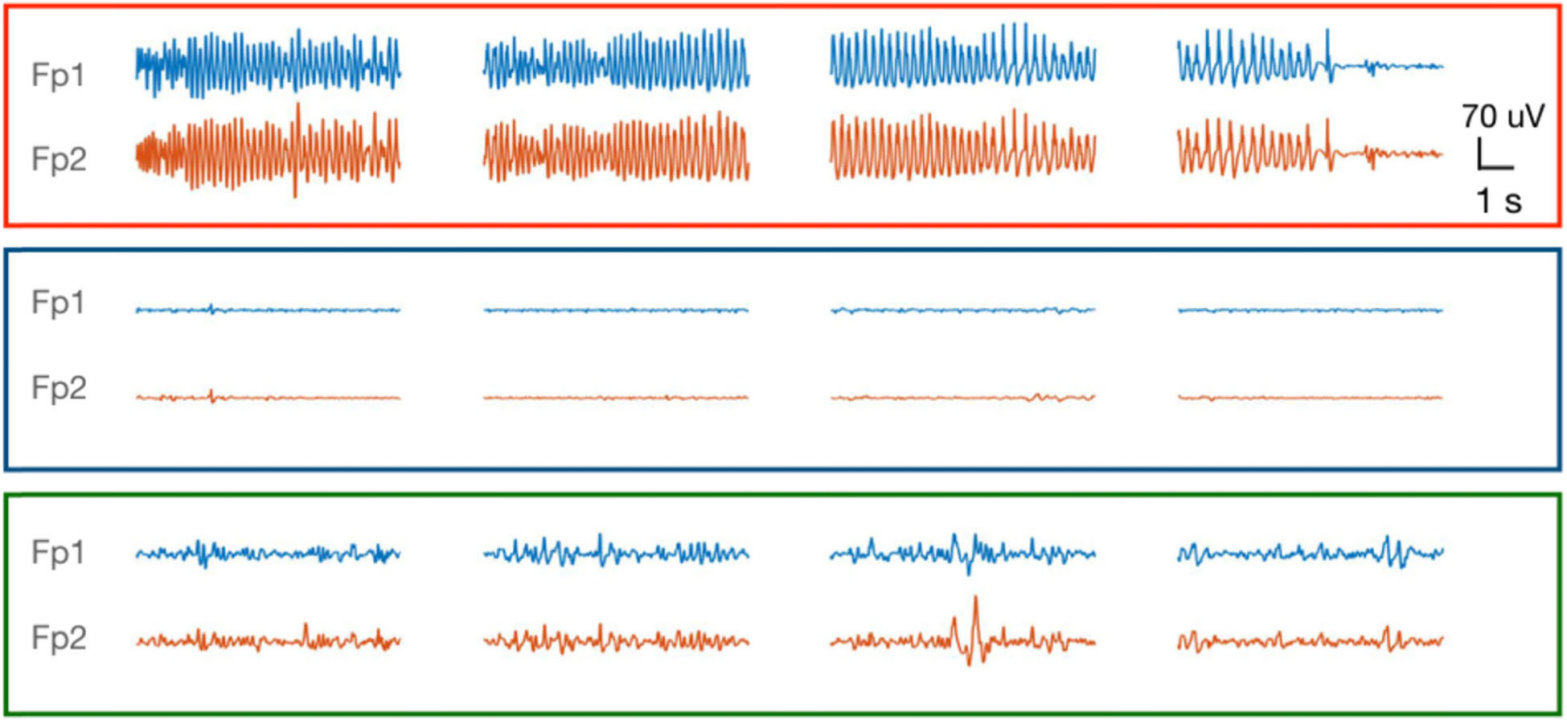
Induced seizure (upper panel) and immediate postictal state (middle panel) EEG epochs of 10 seconds in an ECT patient. Note the flattening of the EEG immediately after the seizure. EEG activity gradually returns after approximately 15 min (lower panel). This EEG evolution is very similar to post-ictal patterns after spontaneous epileptic seizures. Data from an ECT patient treated in Rijnstate Hospital

We propose to study postictal phenomena after ECT-induced seizures. In ECT patients, postictal electroencephalographic (EEG) evolution (Fig. 1) and clinical postictal phenomena are similar to those in patients with epilepsy [8, 9]. The planned and standardized nature of ECT-induced seizures allow us to systematically examine various characteristics of the postictal state (i.e. clinical manifestations, EEG features, cerebral perfusion), including potential effects of interventions. This is a unique model, in a controlled clinical setting, to phenomenologically validate the hypothesis of vasoconstriction-mediated postictal phenomena derived from animal research. The aim of the model is to reap benefits for both psychiatric and epilepsy patients and bridge the gap between both specialisms in neuroscience.

#### Pathophysiology of postictal phenomena is poorly understood

The mechanisms of postictal phenomena, in ECT as well as in epilepsy, are poorly understood and effective treatment is not available. Described major pathophysiological mechanisms involved in the postictal state include changes in extra- and intracellular ion concentrations (e.g. neurotransmitter depletion, decreased extracellular calcium), active inhibition, neurovascular decoupling, blood brain barrier dysfunction, and cerebral perfusion changes [6, 8]. While it has been suggested that neuronal exhaustion is a prominent mechanism in the seizure termination process, neurons are able to generate action potentials even after long periods of activation [6, 10]. The best management of postictal phenomena is still uncertain. In the absence of prophylactic treatment aside from anti-epileptic agents, current treatments of severe postictal symptoms vary from symptomatic symptom suppression with sedatives and triptans to antipsychotic medication [7]. More research is needed to gain insight in the pathophysiology of the postictal state and to identify effective treatment targets.

#### Vasoconstriction-induced hypoperfusion as candidate mechanism

**Fig. 2:**
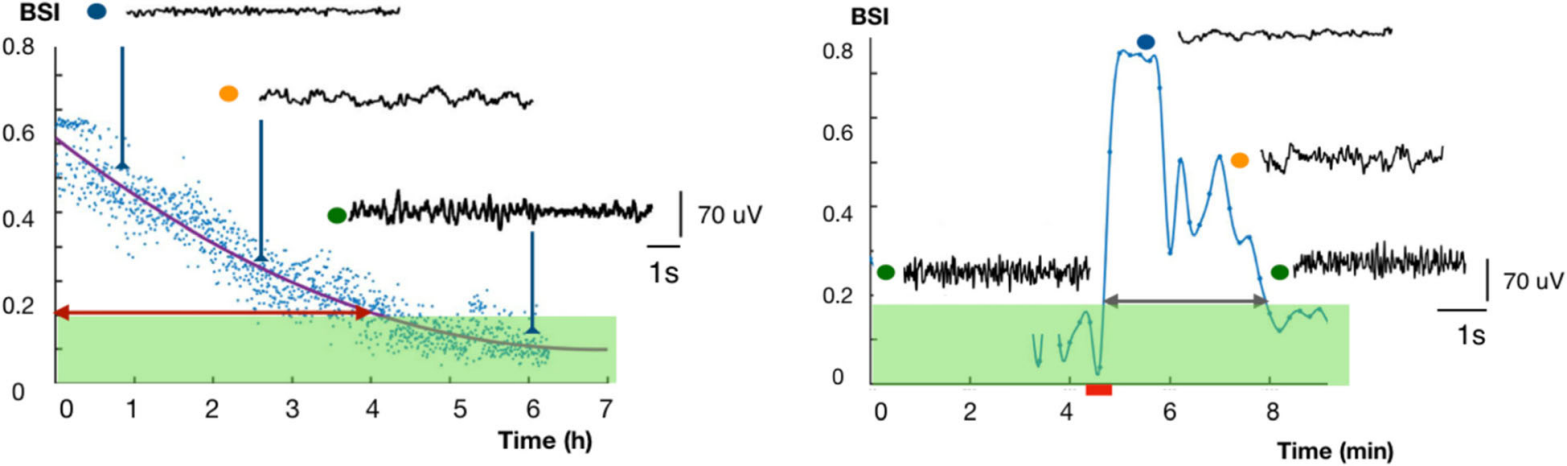
Left: EEG-recovery from global ischaemia in a patient after cardiac arrest (*t* = 0). The nearly iso-electric EEG (blue dot) evolves to a diffusely slowed pattern (yellow dot) to a normal pattern (green dot). Right: EEG traces in a patient with a generalized seizure (red bar, start around *t* = 4.5 min). After the seizure, the EEG is nearly iso-electric (blue dot) and evolves to a diffusely slowed pattern (yellow dot) to a normal pattern (green dot). While the time courses are different in these examples (hours versus minutes), EEG patterns are similar. Only channel P4-O2 is plotted. Data from MST hospital

In 2016, substantial evidence was presented for an alternative mechanism of the postictal state: seizure-induced postictal vasoconstriction with hypoperfusion and hypoxia [11, 12]. Postictal local hypoperfusion had already been observed in patients with temporal lobe epilepsy since the early 1990s [13–15]. Recently, systematic measurements in various commonly used animal models revealed a consistent drop of local blood flow of approximately 50% after brief hippocampal seizures, with a consequent decrease of local partial oxygen pressure. The hypoperfusion and hypoxia lasted more than 1 h postictally. In epileptic patients, equivalent local perfusion deficits were demonstrated with perfusion-weighted MRI, 1 h after seizures. The severity of hypoperfusion correlated with seizure duration, both in rats and patients. Sustained hypoperfusion was associated with local vasoconstriction after electrically induced seizures in rats and after seizure-like activity in acute brain slices [11, 12]. Additionally, in ECT patients, the postictal EEG evolution shows the same characteristics as EEG evolution in patients recovering from cerebral ischemia (Fig. 2) [16]. Both in ECT and epilepsy patients, cerebral hypoperfusion may explain postictal clinical phenomena, because of the similar symptoms of postictal paresis, confusion, psychosis, and cognitive deficits. Moreover, EEG in the postictal period presented as similar to what was observed in patients with transient focal or global ischaemia [16]. This similarity was visible in EEG- and perfusion-recovery (Fig. 2).

#### Acetaminophen and calcium-antagonists reduce postictal hypoperfusion in rats

Multiple mechanisms are established in neurovascular coupling, with activation of L-type calcium channels as a final common pathway. Cyclooxygenase (COX)-2 inhibitors, acetaminophen, and calcium-antagonists were tested to prevent vasoconstriction and hypoperfusion. Indeed, acetaminophen (a partial COX-inhibitor) and nifedipine (a calcium-antagonist) prevented postictal vasoconstriction, hypoperfusion, and subsequent tissue hypoxia [11, 12]. The effect was dose dependent. Prostaglandins were also known to cause vasodilation but—paradoxically—inhibiting the COX-enzyme reduced postictal vasoconstriction and hypoxia. Acetaminophen was more effective in reducing postictal phenomena than other COX-inhibitors, indicating the involvement of currently unknown mechanisms. Exposure to COX-2 inhibitors, acetaminophen and calcium-antagonists did not alter seizure duration, indicating that effects were attributable to a neurovascular mechanism and not via seizure-altering mechanisms [17, 18]. Acetaminophen was only effective if administered before seizure onset, whereas nifedipine also prevented vasoconstriction administered during the postictal state. To further substantiate the possible mediating role of COX-2 in postictal hypoperfusion and hypoxia, experiments were repeated in COX-2 knock-out mice. While local severe hypoxia was observed in control mice, mutants remained normoxic [12].

#### Preventing hypoperfusion reduces postictal clinical phenomena

In animal models, hypoperfusion and hypoxia were associated with clinical postictal phenomena [11, 12]. A postictal recognition memory task was used to compare the influence of acetaminophen and nifedipine treatment in rats. The inability to form new memories, 45 min after the seizure, was recorded in the absence of treatment. Rats receiving acetaminophen or nifedipine before seizures reduced postictal behavioural symptoms to the level of controls. Also, postictal motor deficits in rats as measured by the *hanging bar test* were significantly reduced after treatment with nifedipine [12].

#### Studying effects of acetaminophen and calcium-antagonists in human seizures

Motivated by the findings in animal models, we aim to systematically study the putative role of acetaminophen and nimodipine in vasoconstriction-mediated hypoperfusion and hypoxia on postictal phenomena in humans. In the SYNAPSE study, patients receiving ECT-induced seizures as standard care for severe often treatment-resistant depression will be studied. We will systematically investigate postictal EEG and clinical parameters before, during and after seizures. Various structural and functional MRI parameters will be measured during the postictal period, with additional baseline (before start of ECT) and follow-up (after finishing the ECT course) measurements. If results support that postictal hypoperfusion is an important contributor to postictal phenomena that can be alleviated with vasodilatory treatment, we will identify the first treatment targeting postictal manifestations in humans. This may decrease the burden of ECT for patients with severe depression, and possibly also the burden of the postictal state in patients with epilepsy.

### Objectives {7}

Primary research question:

- Does prior treatment with acetaminophen or nimodipine influence ‘postictal EEG recovery time’ after ECT-induced seizures?

Secondary research questions:

- How does prior treatment with acetaminophen or nimodipine influence postictal hypoperfusion on ASL-MRI after ECT-induced seizures?
- How does prior treatment with acetaminophen or nimodipine influence the clinical ‘time to orientation’ after ECT-induced seizures?
- Are clinical and EEG postictal phenomena associated with ASL-MRI measures of brain perfusion?
- Is influence of acetaminophen or nimodipine on postictal phenomena—assessed with clinical, EEG, and ASL-MRI measures—associated with neurocognitive functioning after the ECT course?

Hypothesis:

Our hypothesis is that acetaminophen or nimodipine will reduce the ‘postictal EEG recovery time’ compared to control. Second, we suppose that acetaminophen or nimodipine will decrease ASL-MRI measures of postictal hypoperfusion compared to control. Third, we expect acetaminophen or nimodipine to reduce clinical time to orientation compared to control. Fourth, we hypothesize to find significant correlations between the established postictal phenomena on clinical, EEG, and ASL-MRI measures. Lastly, we expect to find significant correlations between higher scores of postictal phenomena—assessed with clinical, EEG, and ASL-MRI measures—and more impaired neurocognitive functioning of patients after the ECT course.

### Trial design {8}

**Fig. 3:**
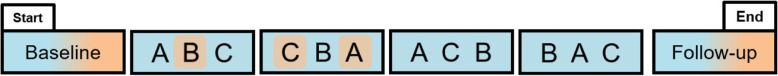
Schematic representation of an ECT course with the intended interventions (*n* = 12) and clinical (*n* = 15), EEG (*n* = 15), and MRI (*n* = 6) measurements. Every patient is randomized to four times a sequence of three conditions (A, B, and C) for three consecutive ECT sessions. A scheme consisting of 12 sessions is depicted, which will be the maximum and intended included number of ECT sessions, save early discontinuation of ECT because of clinical reasons. Baseline includes the ECT titration session (*t* = 0). Blue highlights indicate EEG and clinical measurements, which will be done at baseline, shortly before, during, and after each ECT session, after finishing the total index ECT course, and at 3 months follow-up. Orange highlights indicate MRI measurements (i.e. arterial spin labelling and others), which will be performed at baseline, once in every treatment condition (A, B, and C) directly after the ECT session, after finishing the index ECT course, and at 3 months follow-up

To realize our primary objective, we will conduct a clinical trial with a three-condition crossover design, randomized treatment allocation, open label treatment, and blinded outcome assessments (PROBE design) [19]. The trial is designed in a superiority framework. This indicates that patients will receive one of three treatment conditions prior to each ECT session. Sequences of three conditions will be determined a priori (Fig. 3). Patients will be randomized to a sequence prior to every consecutive period of three ECT sessions. The number of periods (i.e. a sequence of the three conditions) per patient will depend on the total number of ECT sessions for that patient, with a maximum of 4 periods (=12 ECT sessions). Baseline measurements include the first ECT titration session. The primary outcome measure is collected before, during, and directly after each ECT-induced seizure.

## Methods: participants, interventions, and outcomes

### Study setting {9}

This study will take place at the ECT Expertise Centre of the Department of Psychiatry, Rijnstate Hospital, Arnhem, The Netherlands (i.e. a large general teaching hospital with a catchment area of 650,000 inhabitants). In Rijnstate, 1400 ECT sessions take place annually, of which approximately 700 are in ECT-naive patients. The Department of Clinical Neurophysiology of Rijnstate Hospital supports the EEG measurements during this study.

#### ECT procedures

ECT sessions will be administered two times per week, according to the standard protocol of the Netherlands Psychiatric Association (NVvP) [20]. Patients will be anesthetized with etomidate (0.1–0.2 mg/kg body mass), muscle relaxation will be secured with succinylcholine (0.5–1 mg/kg body mass), and appropriate oxygenation (100% oxygen, positive pressure) will be applied until the resumption of the patient’s spontaneous respiration. Preferably, standardized procedures will be used. Start of the index ECT course will be with right unilateral electrode placement (RUL) and a switch to bifrontotemporal (BL) will be done if six RUL ECT sessions appear ineffective or to left unilateral (LUL) in case of severe postictal confusion. Dosage determination will be done by using a dose-titration method—followed by 6 times seizure threshold (ST) in RUL/LUL ECT and 2.5 times ST in BL ECT—or by an age-based dosing method. The used pulse width will be 1.0 ms. If needed, post-ECT sedation will be administered by using midazolam, haloperidol or propofol, to provide comfort and safety to the patient. The ECT course will be terminated if complete remission will be reached, if no further improvement will be seen in two ECT sessions, or if no improvement will be present after a minimum of ten BL ECT sessions. The treating psychiatrist and anaesthesiologist may decide on these treatment variables otherwise based on the clinical conditions. All ECT variables will be recorded for use in analysis.

### Eligibility criteria {10}

#### Inclusion criteria

In order to be eligible to participate in this study, a subject must meet all of the following criteria:

- Adulthood (age > 17 years);
- Current clinical diagnosis of depressive episode (unipolar, bipolar, schizoaffective) as determined by the Mini International Neuropsychiatric Interview (MINI);
- Willingness and ability to give written informed consent and willingness and ability to understand, to participate and to comply with the study requirements;
- Indicated for ECT, according to the Dutch guideline for electroconvulsive therapy (e.g. medication resistant depressive episode, history of successful treatment with ECT, or preference of the patient) [20].

#### Exclusion criteria

A potential subject who meets any of the following criteria will be excluded from participation in this study:

- Known adverse or allergic reactions to acetaminophen or nimodipine;
- Chronic use of acetaminophen, calcium antagonists, or non-steroidal anti-inflammatory drugs (NSAIDs) that cannot be interrupted for less than 2 days before the ECT session;
- Contraindications for MRI (e.g. ferromagnetic implants, pacemakers, claustrophobia). In that case, a participant can be included in the trial without undergoing MRI;
- Contraindications for EEG (e.g. eczema, dreadlocks, intolerability of the EEG procedure).

### Who will take informed consent? {26a}

Oral and written informed consent will be obtained by one of the coordinating researchers or the dedicated research nurse. First, the treating psychiatrist will bring the study to the patient’s (and their relatives) attention. Next, patients will be informed about the procedure by the coordinating researchers or the research nurse. Patients will have at least 3 days to freely decide whether to take part in the study. All remaining questions will be addressed before taking written consent.

### Additional consent provisions for collection and use of participant data and biological specimens {26b}

No additional consent provisions for biological specimens are used, since no specimens will be collected.

### Interventions

#### Explanation for the choice of comparators {6b}

The intervention contrast is acetaminophen vs. nimodipine vs. no additional pharmacological intervention (i.e. a single dose of water). We chose these interventions because of the demonstrated efficacy of acetaminophen and calcium antagonists in rats [12]. Nimodipine instead of nifedipine will be applied, because of its known vasodilatory effects specific to the human brain and profoundly less systemic effects [21].

#### Intervention description {11a}

Apart from the study interventions, standard treatment protocols are utilized [20].

**Fig. 4:**
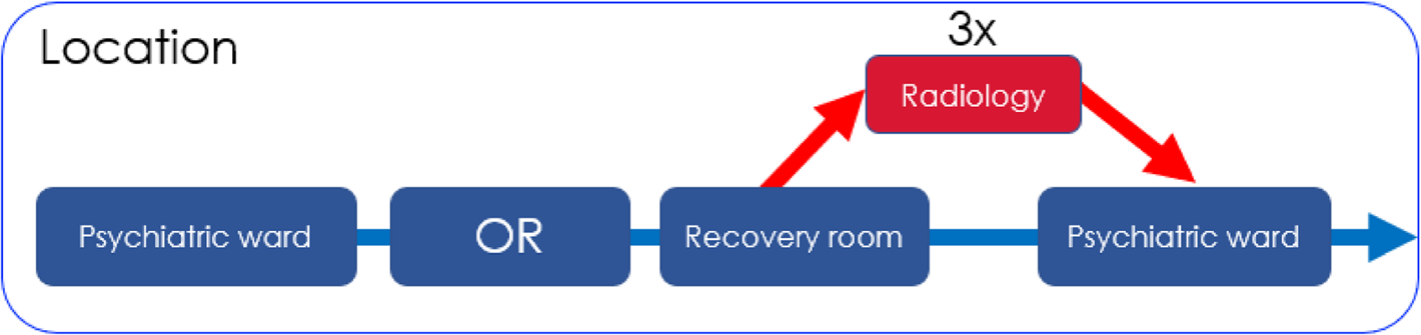
Schematic depiction of the route of stay of patients on the day of an ECT session. Two hours before ECT, patients receive one of the interventions or control on the psychiatric ward. Next, ECT is performed in the operation room (OR). Afterwards, patients are transferred to the recovery room and adjacently back to the psychiatric ward. This route does not differ from standard treatment. Specifically for SYNAPSE, after 3 of the ECT sessions, patients will be transferred directly to the radiology department for the MRI outcome measures before returning to the psychiatric ward

Each patient will receive a single dose of water (50 cc) minimum 2 h, maximum 3 h before the ECT session with either 1 tablet of 1000 mg of acetaminophen, or 2 tablets of 30 mg nimodipine. A minimum of 2 h is required because of the risk of regurgitation during the anaesthetic procedure and the pharmacokinetic properties of both interventions. In the control condition, participants will receive the single dose of water and no tablet. Patients will receive the interventions on the ward. In Fig. 4, the consecutive locations to where patients will be transferred, before and after the study-procedure, are depicted.

#### Criteria for discontinuing or modifying allocated interventions {11b}

Apart from the standard escape medication in ECT, no additional escape drugs are included in this trial. In clinical ECT practice, acetaminophen serves as first line medication in case of (severe) headache or myalgia within the first hours after ECT. Patients may take acetaminophen on the day after the ECT session, but no later than 48 h before the next ECT session. Therewith, due to the *T*_max_ of 2 h and half-life of 4 h, the influence of acetaminophen on the next ECT session is minimized. If participants need pain relief within 48 h before the next ECT session, this ECT session will not be included in the study. Other options for pain relief are considered not to be suitable because of possible effects on cerebral perfusion (NSAID’s, triptans) or increased risk of confusion (opioids) [22].

In case of profound side effects with the use of nimodipine (< 1%), in particular severe symptomatic hypotension, patients can be withdrawn from this drug condition; participation can continue for acetaminophen and the no-treatment condition.

In case of a serious adverse event causing a life-threatening situation or death attributable to our study interventions, the study will be terminated prematurely. After the ECT courses of ten included patients, an evaluation regarding unexpected deviations of effectiveness, observed (severe) side effects or other (severe) adverse events in the SYNAPSE group will be performed and compared to a matched control ECT patient group, without unblinding the interventions. If clinical effectiveness will be significantly lower or adverse events will occur significantly more prevalent in SYNAPSE-patients compared to regular ECT patients, the study will be terminated as well.

#### Strategies to improve adherence to interventions {11c}

All treatment conditions are supplied in the hospital under supervision of the researchers. Study medication is treated according to the hospital’s pharmacist quality standards, including storage, temperature monitoring, and a drug accountability log.

#### Relevant concomitant care permitted or prohibited during the trial {11d}

The chronic use of acetaminophen, calcium antagonists and non-steroidal anti-inflammatory drugs (NSAID’s) is prohibited during participation in this study, except acetaminophen as escape medication directly after the ECT session and not within 48 h prior to a following ECT session. Concomitant psychopharmacological medication and medication for other existing indications (e.g. physical conditions) are permitted and will be analysed for confounding effects.

#### Provisions for post-trial care {30}

An insurance policy is covered for enrolled participants by Rijnstate hospital. The insurance covers damage that may be unintentionally inflicted by participating in this study. This applies to harm that was caused during participation in the study up to four years after participation. The insurance covers damages in line with national regulations of medical-scientific research, which can be found on the website of the ‘Centrale Commissie Mensgebonden Onderzoek’ (www.ccmo.nl).

### Outcomes {12}

#### Primary outcome measure

**Fig. 5:**
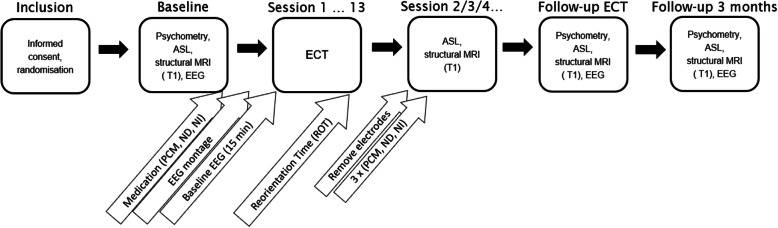
Schematic representation of time schedule and applied measures during SYNAPSE. At baseline, the psychometry battery, EEG and MRI measurements are taken. During and after a maximum of 13 ECT sessions (the first session will be without intervention; in the other 12 ECT sessions, the interventions are applied), intermittent ROT (interval: 5 min) and continuous EEG measures are recorded. Within 1 h after 3 ECT sessions, MRI will take place (one for each intervention). The psychometric measures, EEG, and MRI measurements will be repeated at follow-up within 2 weeks after completion of the ECT course and after 3 months. ROT, reorientation time. ASL, arterial spin labelling. PCM, paracetamol (acetaminophen). ND, nimodipine. NI, no intervention. EEG, electroencephalography. ECT, electroconvulsive therapy

The primary outcome is ‘postictal EEG recovery time’, defined as the time interval between the induction of the seizure and return to the pre-ECT (baseline) EEG, quantified with a modified version of the *temporal brain symmetry index* [23, 24]. An exponential function will be fitted to the data, which will yield a time constant. This provides a quantitative and robust metric of EEG background evolution over time, quantified in minutes. Continuous EEG will be assessed at baseline, during, and until 1 h after each ECT session, as well as after completion of the total ECT course and at 3 months follow-up (Fig. 5).

EEG will be measured with a full lead of 20 electrodes (including ground electrode) in three of the ECT sessions, to gather more extensive data for further research. To reduce the time load and costs, in the other ECT sessions, a lesser amount of 11 electrodes will be used, which still will be sufficient to analyse the primary outcome measure.

Apart from answering our research questions on effects of treatment with acetaminophen and nimodipine on postictal hypoperfusion measures, we will investigate and characterize ictal and postictal EEG dynamics during and after ECT sessions as well as the total ECT course. Targeted future research questions address associations between ictal or postictal EEG phenomena and clinical effects of ECT [25], EEG network dynamics during and after seizures and evolution of excitation-inhibition ratio [26].

#### Secondary outcome measures

##### MRI

Pseudo-continuous arterial spin labelling (pCASL) MRI and structural MRI will be used as MRI-measures to capture a change in cerebral perfusion just after ECT seizures and after the completed ECT course compared to baseline. MRI measurements will be performed on a 3T MRI scanner (Philips Medical Systems, Eindhoven, Netherlands). Postictal hypoperfusion can reliably be detected within 1 h after seizures [12]. Though not one of our research questions directly related to the hypothesis, we will additionally gather T1-weighted MRI data to examine volumetric changes, resting-state functional MRI (RS-fMRI) to measure functional organization and connectivity between brain areas, and diffusion tensor imaging (DTI) to measure diffusion in the brain to estimate the axonal organization. The goal of these additional imaging is to gather data for further research of mechanisms of mood disorders, ECT efficacy and side effects. All MRI measurements will be acquired at baseline, within the first hour after three ECT sessions (one after each of the 3 interventions), after completion of the total ECT course, and at follow-up after 3 months (Fig. 5).

##### Clinical measures

The *postictal reorientation time* (ROT) will be used to indicate when patients are clinically reoriented after the ECT session. Five questions regarding orientation to person, place, and time will be asked repeatedly within an interval of five minutes after administration of the ECT stimulus [27]. Patients are scored as reoriented if 4 out of 5 questions are answered correctly or if a maximum of 90 min is achieved. If the patient is not reoriented at 90 min, a score of 100 min will be noted. The time between the ECT stimulus and reorientation will be expressed in minutes (i.e. the ROT). This measure has been proven to give a robust indication of clinical reorientation [27].

The incidence and severity of postictal headache, nausea and myalgia will be measured by using a visual analogue scale (VAS) [28]. This inquiry will be performed at baseline and at the first moment of full reorientation or at the cut-off of 90 min, as measured by the ROT.

Before start of ECT, after the completed total ECT course and at three months’ follow-up, neurocognitive functioning will be assessed using the Montreal Cognitive Assessment (MoCA), Rey Auditory Verbal Learning Test (RAVLT), category fluency (CF), letter fluency (LF), digit span testing, STROOP task, Trail Making Test A and B (TMT A/B), and Subjective Assessment of Memory Impairment (SAMI). These tests assess multiple cognitive domains (i.e. general cognitive functioning, executive functions, anterograde verbal learning, attention and subjective memory) and have frequently been used in ECT research [29–31].

### Participant timeline {13}

Participants will be informed, asked informed consent and included during a regular consultation in the outpatient Department of Psychiatry in Rijnstate Hospital. If a patient is included, an additional appointment will be made for baseline measurements (Fig. 5). Outcome measurements during and after the ECT sessions do not require additional visits, because these are integrated in the patient care as usual. After completion of the ECT course, the patients will be asked to participate in two follow-up appointments of this study, one within 2 weeks after the end of the total index ECT course, and another one after 3 months. Preferably, these appointments will be coupled to regular outpatient follow-up visits to the treatment team.

### Sample size {14}

Power calculations in humans are hampered by the lack of clinical data on effects of acetaminophen and nimodipine on recovery after seizures. For the sample size calculation, we used G*Power [32]. Assuming an effect size of 0.25, a type 1 error rate of 5%, a correlation of 0.4 between measurements, and 12 included ECT sessions per patient, a total of approximately 33 patients will be needed to achieve a power of .80. This calculation is based on a MANOVA within factors model with repeated measurements, considering a conservative correlation between measurements. In Rijnstate, recruiting patients completing this sample size is feasible in approximately two years (although the COVID-19 crisis will lead to some delay).

### Recruitment {15}

Each patient suffering a depressive episode and who is indicated for ECT will be screened by their treating psychiatrists for the inclusion criteria. If possible, patients and their relatives will be asked to consider participation after a thorough informed consent process. Afterwards, a researcher will explain the procedure and conditions for participating in the study again and more intensively. Every healthcare worker in the Department of Psychiatry of Rijnstate Hospital is educated in the inclusion and exclusion criteria for this trial to inform potential patients and their relatives to maximize inclusion.

### Assignment of interventions: allocation

#### Sequence generation {16a}

Randomized counterbalanced allocation to treatment sequences will be done by an independent team at the hospital’s clinical trial office, using the computer program Research Manager. There are six possible combinations of the three interventions. In total, four sequences will be generated.

#### Concealment mechanism {16b}

The allocation sequence will be concealed in a closed closet until assignment of the participant, not accessible by the investigator performing inclusion.

#### Implementation {16c}

The allocation sequence will be generated by an independent team at the hospital’s clinical trial office. Patients are enrolled by the treating psychiatrists. The researchers or the research nurse acquire informed consent. Interventions will be implemented by one unblinded physician-researcher following the assigned order.

### Assignment of interventions: blinding

#### Who will be blinded {17a}

In this PROBE-design trial, one unblinded physician-researcher will be responsible for supplying the allocated interventions, which will be given to the patient by one unblinded nurse. The other researchers (i.e. the researchers that collect the outcome measures) and the treating psychiatrists will be blind to the treatment allocation. Participants will not be blinded.

#### Procedure for unblinding if needed {17b}

In case of a serious adverse event, the unblinded investigator will inform the treating physician about the treatment allocation immediately.

### Data collection and management

#### Plans for assessment and collection of outcomes {18a}

Baseline psychometric measurements, EEG and MRI will be acquired before the first ECT session. Apart from the psychometry conducted to assess cognitive outcome, the shortened version of the Mini Neuropsychiatric Interview (MINI) and Hamilton Depression Rating Scale (HDRS) will be used for inclusion and repeated HDRS measures for the follow-up of treatment efficacy of the ECT course. Validated Dutch versions of all psychometric measurements are available and will be used. Assessors of psychometric measures are trained by experienced neuropsychologists. The obtained psychometric characteristics are described in Table 1. Directly after administration of the ECT stimulus, in an interval of 5 min, clinical Reorientation Time (ROT) will be assessed. After full reorientation or at cutoff of 90 min, the VAS scales are assessed. The other psychometric scales will be assessed at the timepoints described in Fig. 5. Data collection forms are presented in the appendix.

**Table 1:** Reliability and validity of the psychometric measurements to be used in SYNAPSE

Name of test	Test-retest reliability (*r*)	Construct Validity (*r*)
Trail Making Test (TMT)		
Part A	0.79 [33]	0.63 [34]
Part B	0.89 [33]	0.59 [35]
Stroop Colour-Word Test (Stroop)		Sufficient [36]
Word card	0.83 [37]	–
Colour card	0.74 [37]	–
Word colour card	0.67 [37]	–
Auditory Verbal Learning Test (AVLT)		
Total correct	0.80 [38]	0.58 [39]
Delayed recall	0.83 [38]	0.15 [39]
Letter fluency	0.78 [40]	0.55 [40]
Category fluency	0.92 [41]	Satisfactory [36]
Hamilton Depression Inventory	0.81–0.98 [42]	Adequate [42]
Montreal Cognitive Assessment (MoCA)-Dutch	0.63–0.69 [43]	–
Mini International Neuropsychiatric Interview (MINI)-major depressive disorder	0.87 [44]	–

The online and certified internal Rijnstate system (i.e. Research Manager®) will be used for data storage, using secure access codes. Regular back-up procedures will be applied.

EEG and MRI data will be collected with certified hospital systems and stored in hospital and/or university databases, using secure access codes. Regular back-up procedures will be applied.

#### Plans to promote participant retention and complete follow-up {18b}

Participant retention is promoted by close follow-up during and after the ECT course. For the follow-up appointments after completion of the ECT course, telephone contact is maintained by one of the researchers. Detailed lists of participants and contact information will be handled by the research nurse, who is responsible for scheduling the follow-up assessments. Preferably, follow-up appointments are coupled to regular visits as part of the patient care as usual.

#### Data management {19}

Rijnstate hospital has its own security and storage system of MRI data. MRI data will also be stored on an external and password-protected hard disk and on an access-restricted server of the University of Twente and the Amsterdam University Medical Centres, location AMC. EEG data will be checked for data quality after acquisition and backed-up weekly via an encrypted gateway to an access-restricted server of the University of Twente. Clinical evaluations included in the case record form will be stored in a locked room in a locked cupboard. Data in the case record forms will be digitalized in Research Manager® with the paper forms stored as original backup. Details can be found in the standard operating procedures (SOPs) as part of Rijnstate hospital’s data management plan, as well as the data management plans of the University of Twente and Amsterdam University Medical Centres.

#### Confidentiality {27}

Personal information of participants will be encrypted and password-protected, to which only the study coordinators and principal investigator will have access. Sensitive patient information will be stored in the secured hospital system. Two physical folders will be kept separately, one for anonymous patient data, the other including personal patient data (i.e. informed consent form, interventions assignment and consecutive administrations). Case record forms will be kept anonymous, with specific study numbers, which cannot be connected to the patient directly. After completing the study, unblinding of the interventions will take place and coupled to the anonymous case record forms to complete the database. Personal patient data will never be made public.

### Plans for collection, laboratory evaluation, and storage of biological specimens for genetic or molecular analysis in this trial/future use {33}

In this study, no biologic specimens are collected.

### Statistical methods

#### Statistical methods for primary and secondary outcomes {20a}

First, descriptive statistics will be performed to the relevant variables in each research question. Continuous variables will be presented as mean ± SD, or as median and interquartile range, as appropriate. Data will be presented in tables and figures. There will be assessments of normality and checking for outliers. A Little’s MCAR test will be performed to determine if data is missing at random.

By means of a mixed model analysis repeated measurements analysis (mixed models in R® or SPSS®), we will assess differences in ‘postictal EEG recovery time’ (i.e. the yielded time constant in minutes) between the three treatment conditions. Mixed model analysis takes into account the repeated measures nested in patients and has advantages over other models, since it does not penalize missing data.

We will use ASL-MRI at baseline and directly after the ECT seizures for each intervention to detect relative changes in the whole brain and regional cerebral blood flow compared to baseline. Subsequently, we will test whether these changes are modified by acetaminophen or nimodipine compared to control, measured once per condition. The maximal changes in blood flow will be quantified in mL/100 g/min.

Psychometric measures of cognitive functioning (i.e. change compared to baseline in total scores of MoCA, RAVLT, category fluency, letter fluency, STROOP, TMT A/B and SAMI) after the ECT course will be related to change in ASL blood flow (in ∆mL/100 g/min, voxel-wise analysis), mean difference in ROT (in minutes), and ‘postictal EEG recovery time’ (in minutes). Fixed effects will be age, sex, ECT characteristics (i.e. total number of ECT sessions, bilateral electrode placement expressed in number of sessions, mean dose of stimulus), and the pre- and post-ECT course HDRS scores.

#### Interim analyses {21b}a

As the influence of our interventions on ECT efficacy is largely unknown, an interim analysis will be performed after the inclusion of ten participants to assure expected treatment effectivity. Hamilton Depression Rating Scale scores of patients included in this trial will be compared to those of a similar patient sample (matched for electrode placement, age and sex) derived from an earlier study in Rijnstate with ECT patients (trial registration number: NL24697.091.09). In case of a statistically significant lower rate of remission in SYNAPSE patients, the study will be terminated prematurely.

#### Methods for additional analyses (e.g. subgroup analyses) {20b}

Subgroup and adjusted analyses will be performed to correct for factors including but not limited to age, comorbidity, and comedication use. Corrections for multiple comparisons will be made (i.e. Bonferroni correction or others).

#### Methods in analysis to handle protocol non-adherence and any statistical methods to handle missing data {20c}

Dropouts will be replaced by new inclusions to meet the targeted number of included patients. Missing data will be accounted for in mixed model statistical analysis.

#### Plans to give access to the full protocol, participant level-data, and statistical code {31c}

We plan to give access to the statistical code for our primary outcome measure. Anonymized participant data will be made available at request. Anonymous MRI data will (after analysis of the primary research question) be shared within the Global ECT-MRI Consortium (GEMRIC) to facilitate international research.

### Oversight and monitoring

#### Composition of the coordinating centre and trial steering committee {5d}

Daily coordination of the trial (f.e., coordinating logistics of patient measurements, executing measurements, handling trial medication) is managed by the coordinating researchers. The principal investigator performs daily supervision. The other researchers supervise parts of the study from their respective specialities, f.e. EEG quality control. The Trial Steering Committee meets two to three times a year. The frequency of this supervision ranges from once per week to maximum once per month. There is no Stakeholder and Public Involvement Group (SPIG) involved in this trial. For this single-centre trial, no coordinating centre is needed.

#### Composition of the data monitoring committee, its role and reporting structure {21a}

No Data and Safety Monitoring Board is deemed necessary when considering the current practice in ECT management, the open label conditions, and the negligible added risk of the activities of the study. The accredited Medical Ethical Committee permitted the absence of a data monitoring committee.

#### Adverse event reporting and harms {22}

All adverse events reported spontaneously by the patient or observed by the investigators will be recorded. Serious adverse events will be reported to the accredited Medical Ethical Committee. Details indicating expectedness, seriousness, severity, and causality will be supplied.

#### Frequency and plans for auditing trial conduct {23}

Auditing will take place by an independent monitor. Included in the audit process are checks for informed consent forms, inclusion criteria, and source data validation. The first audit will be scheduled after study completion of the third participant, with yearly audits thereafter.

Plans for communicating important protocol amendments to relevant parties (e.g. trial participants, ethical committees) {25}

Important protocol modifications are communicated without delay to all involved researchers, the Medical Ethical Committee and the monitor. Trial registries will be updated. Any deviations from the protocol will be documented using a breach report form.

#### Dissemination plans {31a}

Approximately 2 years after completion of the trial, results will be communicated to the participants via email. The public and healthcare professionals will be informed via international publications and conference proceedings.

## Discussion

We present a study protocol for a randomized cross-over trial with PROBE-design to investigate effects of acetaminophen and nimodipine intending to reduce postictal phenomena after ECT-induced seizures. We will now discuss several aspects of this study.

### Study design

We will apply a sequential trial with a cross over treatment design, in which each participant will be randomly assigned to a series of intervention conditions. Each participant will serve as their own control. Since we expect considerable interindividual variability of the outcome values, this design optimizes the chance of finding small treatment effects. Because our study population will be representative of usual ECT patients in all different severities, results may be directly applicable to the general ECT population suffering a major depressive episode.

### Outcome measures

Our primary outcome measure will be the speed of postictal EEG recovery, quantified as published previously [23, 24]. We chose this measure because of objectivity, directness, and the assumption of optimal discrimination between groups. The measure is objective, because of computer assisted calculation, off line, after the experiments, and blinded to the intervention allocation. It constitutes of a direct measure of brain functioning, which will be collected continuously. MRI measures of perfusion will be used to substantiate the assumption that a potential treatment effect is associated with modulation of cerebral perfusion. This will also be an objective computer-assisted measure. Furthermore, the clinical ROT will be included as secondary outcome measure. The ROT is highly clinically relevant and a good predictor of cognitive side effects in ECT patients [27, 45, 46]. However, detection of small intervention effects may be hampered by intermittent sampling (i.e. every 5 min) and ceiling effects. Ceiling effects were indeed observed in our pilot experiments, where reorientation according to ROT was achieved hours earlier than EEG recovery (data not shown).

### Interventions

In the experimental studies that gave rise to this trial, various COX-inhibitors and calcium antagonists reduced postictal phenomena, with the strongest effects of acetaminophen and nifedipine [12]. Effects of acetaminophen were established alongside effects of selective COX-2 inhibitors, exerting its effects via the COX-2 dependent vasoconstrictive mechanism. Since acetaminophen has no relevant side effects, is widespread available, and is cheap, we chose to test this drug instead of a selective COX-2 inhibitor. Furthermore, we chose to test nimodipine instead of nifedipine, since nimodipine is known to have less systemic vasodilatative properties and side effects in humans. The highest regular dosages will be used to maximize presumed treatment effects. If an effect will be established, patients with recurrent epileptic or ECT-induced seizures may benefit from (chronic) acetaminophen treatment. Since treatment with calcium-antagonists may even be beneficial if initiated shortly after the seizure [11, 12], patients with epilepsy may eventually benefit from acute postictal ingestion.

### ECT as human model of epilepsy

In this study, the examined ECT-induced seizures will be part of the treatment of patients with a severe depressive episode. The postictal period in ECT-induced seizures and spontaneous seizures in epilepsy patients are clinically and electrographically comparable [4]. Clinical similarities include sensory, motor, or memory deficits, impaired cognition, headache, delirium, or psychosis [5–7]. Similarities in postictal EEG evolution include a period of EEG depression, followed by diffusely slow EEG, evolving towards normal in minutes to hours (see Fig. 1) [8]. Our study design offers an opportunity to systematically study the postictal state, including effects of interventions. This is not feasible in patients with epilepsy due to the erratic nature of seizures. Despite obvious differences with spontaneous seizures, mainly the use of anaesthetic drugs and the presence of major depressive episodes in our population, results may be generalized to patients with epilepsy.

### Logistic challenges

This study will be the first in systematically measuring continuous EEG before, during and directly after ECT-induced seizures with full EEG montage. The procedure of continuous EEG measuring is challenging, because the patients’ journey during our SYNAPSE-ECT sessions will be complicated and will involve measurements at different places within the hospital. During the ECT seizure and the transportations, the researchers will try to minimize artefacts in order to reach a minimum of one artefact-free segment of 5 s during the postictal state. In Rijnstate hospital, the involved operating room and radiology department are on the same floor, and the psychiatry ward is one floor below. At time of the writing of this paper, we have experience with fifteen patients undergoing this whole procedure. We have noticed that it is feasible, although barriers may be present. Due to the flexibility and cooperativeness of all involved professionals and departments, our research protocol has proven to be manageable.

### Possible positive spin off of this study

Our study may have a positive spin off for patients with major depressive episodes treated with ECT. First, these patients generally have low self-esteem and consequently experience uselessness, insignificance, or worthlessness. Participation in a scientific study, which may have advantages for future ECT and epilepsy patients, may lead to a sense of usefulness [47]. Second, patients with major depressive episodes (or other psychiatric disease) often feel stigmatized [48]. Their disease is regarded as ‘non-existent’, as no direct somatic origin can be established. In the SYNAPSE trial, clear EEG and MRI measures of brain functions are collected, to underpin the biological nature of our population’s disease and effects of ECT. This may reduce stigmatization of both the disease and ECT as effective treatment. Close collaboration between neurologists and psychiatrists in this study may contribute to de-stigmatization as well and may bridge the gap between these specialties [49]. Finally, if post-ECT cognitive deficits may ultimately be reduced by our interventions under study, ECT may become more tolerable and acceptable for patients, their relatives and society.

### Strengths and limitations

Our study will have certain limitations. First, we will apply open-label interventions. However, risk of bias is minimized for the primary outcome measure, since EEG analyses will be done off line, blinded to intervention allocation and fully automated. Second, we expect considerable heterogeneity in covariates, such as ECT parameters. Confounding will be minimized by using a sequential treatment design and adjustment in analysis. Finally, due to the complex patient journey during the SYNAPSE trial, significant missing data may be expected, which must be taken into account in our analyses.

In conclusion, SYNAPSE will be the first randomized clinical trial to evaluate effects of acetaminophen and nimodipine intending to alleviate postictal phenomena in depressed patients receiving ECT. If any of the interventions appear to be effective, we will have identified the first effective treatment targeting postictal symptoms. Results may be generalized to patients with epilepsy. Bridging insights from neurology and psychiatry may reduce the burden for patients and their relatives living with epilepsy or treated for severe psychiatric disorders.

## Trial status

Protocol version number 1.7, 27-09-2020

Recruitment started on December 5, 2019

Recruitment will be completed around July 2023

## Abbreviations

ASL: Arterial spin labelling
CF: Category fluency
COX: Cyclooxygenase
ECT: Electroconvulsive therapy
EEG: Electroencephalography
EudraCT: European drug regulatory affairs Clinical Trials
GCP: Good Clinical Practice
GEMRIC: Global ECT-MRI Consortium
HDRS: Hamilton Depression Rating Scale
LF: Letter fluency
MINI: Mini-neuropsychiatric Interview
MoCa: Montreal Cognitive Assessment
MRI: Magnetic resonance imaging
ND: Nimodipine
NI: No intervention
NSAID: Nonsteroidal anti-inflammatory drug
NVVP: Netherlands Psychiatric Association
OR: Operating room
PCM: Paracetamol (acetaminophen)
PROBE: Prospective Randomized Open Blinded End-Point
RAVLT: Rey Auditory Verbal Learning Test
ROT: Reorientation time
SAMI: Subjective Assessment of Memory Impairment
SYNAPSE: StudY of effect of Nimodipine and Acetaminophen on Postictal Symptoms in depressed patients after Electroconvulsive therapy
TMT A/B: Trail Making Test A and B
VAS: Visual analogue scale
